# Case report: angiosarcoma of the pulmonary artery diagnosed as pulmonary artery emboli

**DOI:** 10.1186/s13019-022-02036-w

**Published:** 2022-11-16

**Authors:** Edina Korça, Jad Kanaan, Kálmán Benke, Gábor Veres, Jens Michaelsen, Gábor Szabó

**Affiliations:** 1 Department of Cardiac Surgery, Middle German Heart Centre, University Hospital Halle (Saale), Martin-Luther University Halle-Wittenberg, Ernst-Grube-Str. 40, 06120 Halle (Saale), Germany; 2 Heart and Vascular Center Semmelweis University, Budapest, Hungary

**Keywords:** Angiosarcoma, Emboli, Pulmonary artery, Cardiac tumor

## Abstract

**Background:**

Angiosarcomas are the most common malignant tumors of the heart and great vessels. Late onset and unspecific symptoms are reasons why a diagnosis is made rather late at a time when most tumors have already metastasized. We report a rare case of an angiosarcoma presenting as pulmonary artery emboli.

**Case presentation:**

A 66-year-old patient was initially admitted to the hospital with a STEMI. Days later a successful reanimation due to ventricular fibrillation followed. An emboli of the pulmonary artery was diagnosed as the cause and after a lysis therapy the patient was discharged. A few weeks later the patient suffered an episode of absolute arrhythmia. TTE as well as CT-Scan showed an emboli of the pulmonary artery and a pericardial effusion with compression to the right ventricle. Intraoperative findings, showed an infiltrating tumor of the pulmonary artery, the pulmonary valve, the RVOT, the LA and LV. A resection of the tumor from the pulmonary artery, valve and RVOT was carried out. A new pulmonary valve was implanted with the reconstruction of the RVOT and pulmonary artery. Due to LV infiltration, only a palliative surgical approach was possible. Despite an uncomplicated postoperative course, the patient died at home two months later.

**Conclusion:**

Although a rarity, a tumor of the pulmonary artery should be taken into consideration as a differential diagnosis to pulmonary artery emboli. Development of better diagnostic tools (specific tumor markers) and more effective chemotherapeutic agents is necessary to improve the prognosis of these patients.

## **Background**

Primary tumors of the heart and great vessels are rare, yet they are often life-threatening and require immediate treatment. According to large autopsy studies, 75% of such tumors are benign and 25% are malignant [1].

Atrial myxomas are the most frequently diagnosed benign primary cardiac tumors in adults, and they usually arise from the left atrium. Angiosarcomas are the predominant malignant primary tumors of the heart, with the right atrium being the most common site of genesis [2].

Patients present with nonspecific symptoms such as weight loss, fatigue, or night sweats. The obstruction of blood flow in the right ventricular outflow tract (RVOT) causes dyspnea and other symptoms of heart failure. Patients may also present with arrhythmias and embolisms. In situations of malignant tumors, hemorrhagic pericardial effusion is very common [3].

Imaging techniques such as echocardiography, computed tomography (CT), magnetic resonance imaging (MRI), and 18 F-fluorodeoxyglucose positron emission tomography/computed tomography (FDG-PET/CT) are used to make the initial diagnosis. For final diagnosis, however, histological analysis is required.

Regarding tumors of the pulmonary artery clinical and imaging manifestations may mimic those of pulmonary embolism [4].

The gold standard treatment for malignant tumors is surgical excision of the tumor followed by chemo- and radiotherapy. For benign tumors, a simple resection is typically sufficient, but for malignant tumors, a comprehensive resection with repair or replacement of afflicted tissues is required. If the tumor has already infiltrated the dorsal part of the great vessels and the posterior wall of the left atrium an ex-situ resection of the tumor may be suitable [2, 5].

Patients with malignant cardiac tumors have also been documented to undergo TAH (Total artificial heart) implantation or even heart transplantation as a last option [6].

The prognosis is poor, and patients usually succumb from distant metastases.

## Case presentation

Initially, the patient was admitted to the hospital with a STEMI (ST- Elevation myocardial infarction). Three DES (Drug Eluting Stents) were implanted in the LAD (Left anterior descending artery) and its diagonalis branch. The interventional cardiologists, did not express any suspicion about possible mass lesions in and around the pulmonary artery at the time of coronary artery stenting.

While still in the hospital, the patient developed ventricular fibrillation four days later. ROSC (Return of spontaneous circulation) occurred after a ten-minute resuscitation.

The patient was then transferred to the ICU (Intensive care unit). He was in need of catecholamines, had ventricular tachycardia, and was on invasive ventilation when he was admitted to the ICU. He eventually transitioned to a sinus rhythm after receiving further medication.

A TTE (Transthoracic echocardiography) showed a reduced left ventricular ejection fraction (LVEF) and a dilated right ventricle with signs of acute right heart load. The thorax CT-scan showed a massive bilateral pulmonary embolism. Heparin medication was initiated, and the patient was immediately transferred to a tertiary care hospital’s cardiology department.

After exclusion of an intracranial hemorrhage via cranial CT-scan, a lysis therapy with Alteplase followed. The patient was extubated the next day and weaned off the catecholamines. The following TTE two days after lysis showed a regressive right heart load, slight tricuspid valve insufficiency (TAPSE = 22 mm, PAPdpmax = 43mmHg + 10mmHg CVP) and a slightly reduced LVEF, with the pulmonary valve described as inconspicuous.

The patient was transferred back to the referrer hospital on the third day, with the recommendation for therapeutic anticoagulation and further examination to identify the source of the emboli.

The patient was released home and then proceeded to a rehabilitation clinic for physical treatment after his further stay in the hospital was uneventful.

Twenty days later while in the rehabilitation center the patient got an extreme tachyarrhythmia, as well as acute onset of dyspnea and was taken to the hospital (one and a half months after the STEMI).

An emergency surgery was required due to suspicion of an apical aneurysm with covered perforation of the ventricle, an additional three centimeter pericardial effusion with compression of the right ventricle, and suspected recurrent thrombus in the pulmonary artery and RVOT (Right ventricular outflow tract) (TTE before surgery: TR PPG 40mmHg; TAPSE normal range; pulmonary valve Vmax 2.5 m/s. RV-PA PPG/MPG 24/14 mmHg; thrombus in pulmonary artery and right ventricular outflow tract (3.6 × 8.3 cm)).

**Fig. 1:**
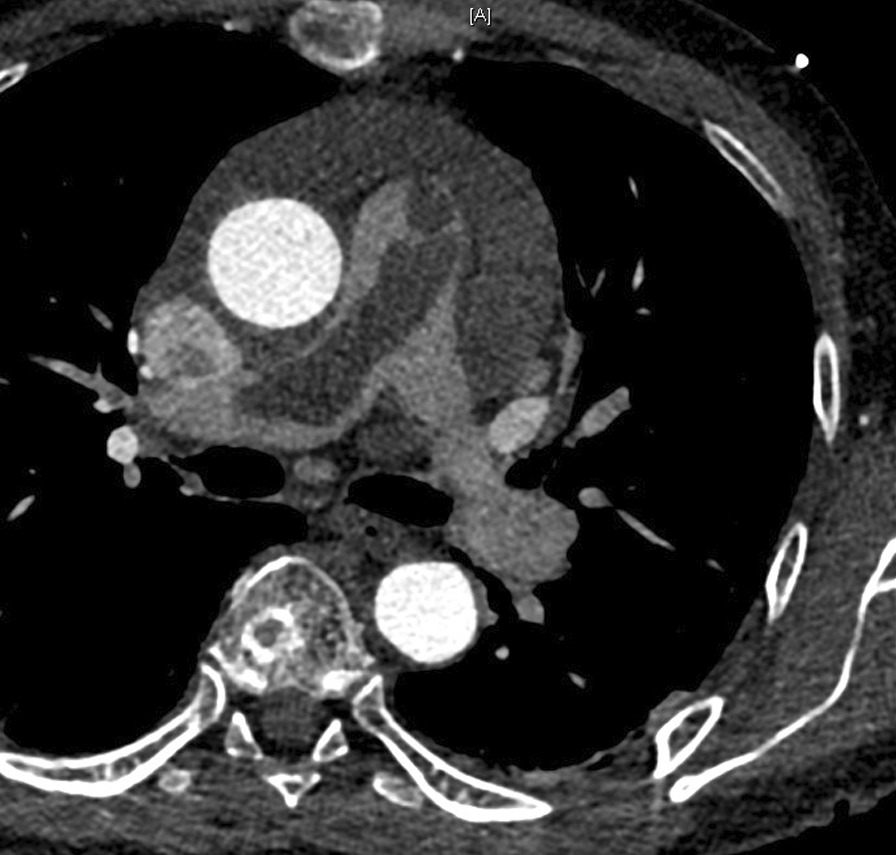
Preoperative CT-scan showing the tumor in the main and right pulmonary arteries

On the same night, a CT-scan revealed a mass in the pulmonary artery, RVOT, and pulmonary valve (Fig. 1). The patient was immediately transferred to a specialized cardiac surgery center for an urgent operation.

For the operation the ascending aorta and both caval veins were cannulated after opening the pericardium, removing 400 ml blood from the pericardial cavity, and heparinization. The patient was then cooled to 33 °C. Two liters of Custodiol solution were used for cardioplegia.

After a longitudinal opening of the main pulmonary artery, a tumor adherent to the lateral wall of the pulmonary artery, covering almost its entire lumen, with infiltration of the pulmonary valve and extension to the RVOT was seen. Resection of the tumor from the lumen of the pulmonary artery together with the lateral wall of the artery, the pulmonary valve, and the infiltrated part of the RVOT followed. After the above-mentioned portions were removed, local metastases were seen on the main right pulmonary artery. Further, the tumor had also infiltrated the dorsal wall of the main pulmonary artery, the upper wall of the left atrium, and the left ventricle. A complete resection was not possible. Further revision of the distal branches of the pulmonary artery was not performed.

**Fig. 2:**
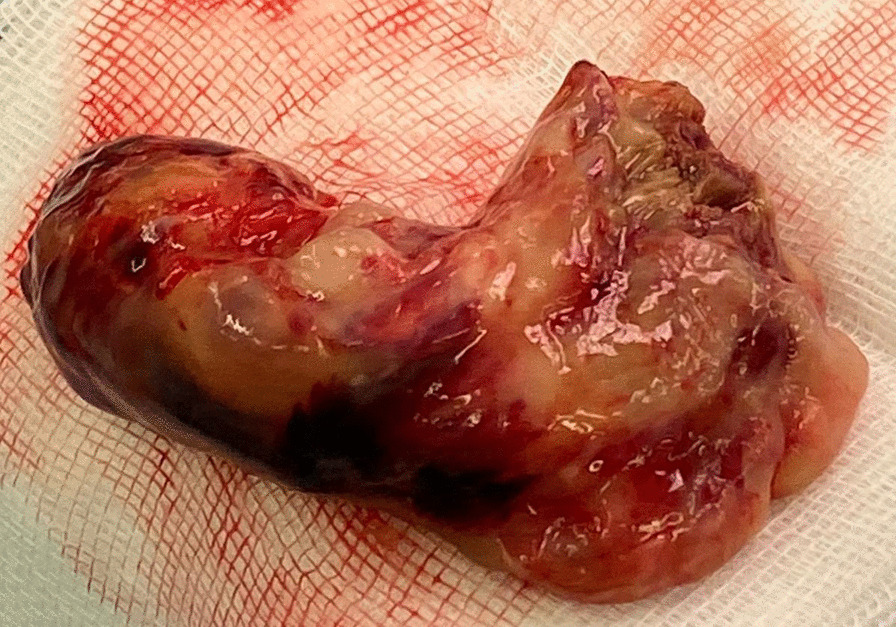
A section of the resected tumor

After that, the RVOT was reconstructed. Seven 2−0 Ethibond pledgeted sutures were placed in the height of the previous pulmonary valve annulus. Following the measurement, a partial implantation of a 25 mm CE Perimount bioprosthesis was performed with the help of the previously inserted pledgeted sutures. A 10 × 4 cm Gore-Tex-Patch was used to reconstruct the RVOT. The patch was sewn up to the level of the pulmonary valve using a 4 −0 Prolene continuous suture. The rest of the pulmonary valve was implanted on the Gore-Tex-Patch using 2 − 0 Ethibond pledgeted sutures. The patch’s upper part was then sewn to the pulmonary artery’s lateral wall to repair it (Fig. 2).

The weaning from cardiopulmonary bypass was uneventful.

Intraoperative TEE (transesophageal echocardiography) showed a good function of the implanted pulmonary valve.

The patient’s postoperative recovery was uneventful. The histological findings confirmed the suspicion of a high-grade angiosarcoma. PET/CT also showed that the tumor had progressed to the left ventricle. Metastases were not discovered. After discussion of the case in our tumor board the patient was scheduled to begin chemotherapy after some recovery time. On the 13th postoperative day, he was discharged and died two months later at home.

## Discussion

Primary cardiac angiosarcoma is a rare tumor commonly found in the right atrium. In a review of cardiac angiosarcomas, Janigan et al. [7] reported right and left atrium involvement in 93% and 7% of cases, respectively.

Goyard C et al. presented a case initially diagnosed as pulmonary embolism. A pulmonary artery sarcoma was detected and later discovered as the patient’s pulmonary hypertension worsened despite his vitamin K antagonist therapy [8].

The likelihood of a tumor in the pulmonary artery was not considered in this case during the initial hospitalization. It would have been feasible to determine the extent of the tumor’s spread if the diagnosis had been made earlier. However, due to signs of cardiac tamponade and obstruction of RVOT at the time of diagnosis, the patient was in need of an emergent operation.

A pulmonary artery tumor should be considered as a differential diagnosis for pulmonary embolism, notwithstanding its rarity.

Experienced cardiologists should evaluate echocardiography findings, giving special attention to the pulmonary artery. After a simple instance of pulmonary embolism, the outcome of therapy can be determined by using echocardiography to estimate right heart load. However, in complicated cases, a control CT following lysis should be recommended. When a tumor is suspected, a CT-scan should be performed.

Although there have been cases of heart transplantation in patients with cardiac tumors reported in the literature, due to a scarcity of organ donors, even with extensive preoperative diagnostics and the exclusion of distant metastases, a heart transplant would not have been an option for the patient.

Regarding the patient’s death in the absence of an autopsy, we can only speculate about possible causes such as further tumor involvement of the left ventricle, displacement of the LAD Stent, ventricular arrhythmia, and so on.

## Conclusion

Although uncommon, a tumor of the pulmonary artery should be considered as a differential diagnosis for pulmonary artery emboli.

To improve the prognosis of these patients, new diagnostic methods (particular tumor markers) and more effective chemotherapeutic agents are required.

## Data Availability

Not applicable.

## Abbreviations

CT: Computed tomography
DES: Drug eluting stents
ICU: Intensive care unit
LAD: Left anterior descending artery
LA: Left atrium
LV: Left ventricle
LVEF: Left ventricular ejection fraction
MRI: Magnetic resonance imaging
PET/CT: Positron emission tomography/computed tomography
RVOT: Right ventricular outflow tract
ROSC: Return of spontaneous circulation
STEMI: ST elevation myocardial infarction
TAH: Total artificial heart
TTE: Transthoracic echocardiography
TEE: Transesophageal echocardiography
